# Novel genetic association with migratory diapause in Australian monarch butterflies

**DOI:** 10.1186/s12862-025-02384-w

**Published:** 2025-05-07

**Authors:** William Hemstrom, Micah Freedman, Myron P. Zalucki, Michael Miller

**Affiliations:** 1 Department of Animal Science, University of California, Davis, Davis, CA USA; 2 Department of Biology, Colorado State University, Fort Collins, CO USA; 3 Department of Ecology and Evolutionary Biology, University of Toronto, Toronto, ON Canada; 4 School of the Environment, The University of Queensland, Brisbane, QLD Australia

**Keywords:** Monarch butterflies, Migration, Genomics, Reproductive diapause, GWAS

## Abstract

**Background:**

Monarch butterflies (*Danaus plexippus*) are a charismatic and culturally important North American butterfly species famous for their unique, dramatic migratory life history. While non-migratory populations of the species are widespread and apparently stable, migratory populations in North America have recently seen declines, prompting concern that the migratory phenomenon in North America may be at risk of disappearing. In contrast, a relatively recently-established monarch population in Australia has rapidly re-acquired a migratory life history following hundreds of generations of residency and successive bottlenecks as the species island-hopped across the Pacific during the late 1800s and early 1900s. The process by which migration re-emerged in Australian monarchs is not currently known.

**Results:**

We raised and sequenced individuals from Queensland, Australia under environmental conditions associated with migration initiation and found strong variance in reproductive diapause, a key migratory trait, between families which was associated with variation at the spectrin beta chain protein *Karst*. This protein is known to be involved in diapause termination in monarchs but has not previously been identified as associated with migratory life history variance. The most strongly associated migratory SNPs are also present at a low frequency in North America, suggesting that the Australian population is leveraging standing variation which persisted across repeated bottlenecks as Monarchs spread across the Pacific.

**Conclusions:**

Our results provide an intriguing example of how the temporary loss of migration—in this case likely over hundreds of generations—may not entail the loss of genetic variation associated with this complex life history strategy.

**Supplementary Information:**

The online version contains supplementary material available at 10.1186/s12862-025-02384-w.

## Background

Migratory species are acutely at risk from global anthropogenic environmental change [1, 2]. The loss of breeding, wintering, or transitional habitats may all cause declines in migrants [3], as may shifts in resource phenology due to seasonal changes which can cause resource availability and species life-histories to fall out of synch [4]. It is therefore not surprising that widespread declines have been observed in many migratory taxa [3, 5, 6].

Population-level adaptability in migratory life-history should therefore be expected to increase long-term population viability by conferring resistance to phenological and environmental changes [7]. Migratory life-history adaptation can occur along three distinct axes: 1) *temporal* adaptation that changes the phenology of migration; 2) *spatial* adaptation that alters migratory pathways and orientation; and 3) *residency* adaptation that shifts populations between migratory and resident life histories. Inter-population variation and adaptation in each of these pathways is well known from nature: temporal changes or variation in migration are well documented in many taxa including birds [8–13], fish [14], mammals [15], and butterflies [16, 17], as are short-stopping, the extension of migratory pathways, or the complete re-direction of migration [18–25] and shifts towards residency or partial migration in birds [7, 26–30], butterflies [31], mammals [32–34], and fish [35].

Adaptation via a transition to residency is of particular interest and concern for the conservation of migratory species. While switching to a resident life history can allow species to avoid the increasing risks of migration and the intrinsically large energetic costs of migration in general [36–38], there is concern that the “extinction” of migration may not be easily reversible in some species. Permanent loss of migratory behavior is problematic because, in general, variation in migratory tendency is expected to increase long-term population persistence [39]. Beyond this, migratory species often provide critical ecosystem services [40, 41] and have cultural and/or ecological significance [42–45] that may not survive the transition to permanent residency.

The loss of migratory behavior is a major concern for monarch butterflies (*Danaus plexippus*) [46, 47]. Specifically, North American migratory monarch populations have experienced pronounced declines in overwintering individuals over the last several decades, sparking fears that those populations may go extinct in the near future [48] (although not all agree [49]). However, many individual butterflies ostensibly from the North American migratory population are now year-round residents in some areas in the southern United States [31], where they can survive due to the year-round presence of introduced tropical milkweeds (e.g. *Asclepias curassavica*, *Gomphocarpus sp., Calotropis sp.*) [50], and there are many other non-migratory monarch populations that are not of any immediate conservation concern [51]. While the species as a whole may therefore not be at risk, their migratory behavior seemingly is. In addition to the damage to the long-term population persistence that could result from the loss of migration in North American populations due to the loss of life-history variation (as discussed above), North American monarchs carry large deleterious genetic loads which are likely buffered by increased effective population sizes and reduced inbreeding facilitated by migration-induced gene flow [52], and the loss of the publicly beloved annual monarch migration and concomitant overwintering would be culturally costly [53].

**Fig. 1:**
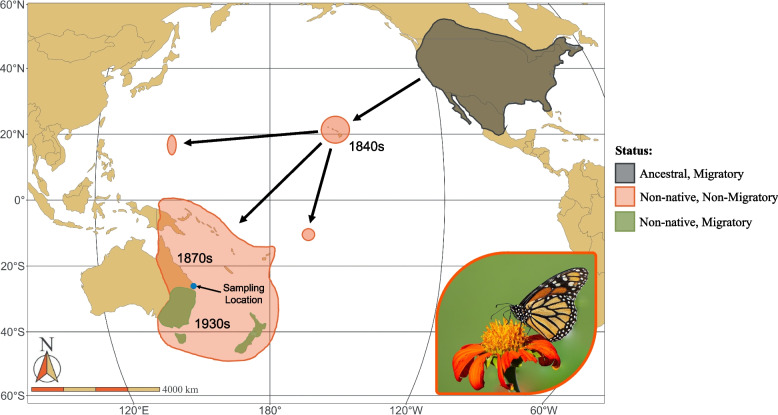
Approximate ancestral North American and introduced migratory and non-migratory ranges for monach butterflies, with approximate introduction dates in the Pacific are noted. Sampling location noted in blue. Photo © William Hemstrom, 2022.

In contrast, there is a population of monarchs in Australia in which migratory behavior has apparently re-emerged after it was lost. This population, which was established sometime before 1871 [54], is the product of a series of sequential introductions that crossed the Pacific Ocean from North America beginning sometime in the early 1840s after the introduction of their host plants to Hawaii and other Pacific islands enabled individuals to survive after being blown from the mainland (Fig. 1) [54–56]. Monarchs are not known to be migratory on any Pacific islands, and no migratory behavior was observed in Australia until the 1930s, when non-reproductive overwintering aggregations were first observed in northern New South Wales (NSW) [57]. These and other individuals were later shown to migrate hundreds of kilometers each year to their wintering grounds from more southerly, inland locations in NSW [57, 58]. Thus, more than 90 years (and hundreds of generations) after expanding out of North America without any evidence for seasonal migration, migratory behavior re-emerged in these populations. Understanding how this happened could provide crucial context around the “extinction” of migration in monarchs and other species.

To better understand how migration re-emerged in Australian monarchs, we studied continuously breeding monarch populations in Queensland. While these populations do not experience substantial seasonal fluctuations in resource availability, and thus do not migrate [57], recent work has shown that reproductive diapause can be induced in some, *but not all* individuals from these populations if they are exposed to a reduction in photoperiod during development [59], a trait that these non-migratory populations do not display in the wild. In brief, reproductive diapause, where individuals delay their reproductive maturation, is a critical and fitness-relevant phenotype for migratory monarchs because it allows them to invest more heavily in lipid storage and increase their average longevity [60–62]. Like other migratory behaviors, diapause is initiated by changes in day length, temperature, and other seasonal fluctuations [63–65] and is thought to be directly controlled by juvenile hormone (JH) titers during development [60, 61, 66].

While diapause is one of many migration-associated traits (such as wing morphology, resting and flight metabolic rates, lifespan, and lipid storage), it in particular is linked extremely tightly to migration. Nearly all wild migratory monarch females are in diapause: previous work has found that between 82 and 100% of female migratory monarch butterflies are non-reproductive [47, 63, 67, 68], and previous rearing trials under typical North American summer breeding conditions have yielded nearly 100% reproductively mature females [63]. These trends are less pronounced in male monarchs, which are therefore not the focus of this study [47, 67]. Given that phenotyping the entire migratory syndrome is challenging, diapause serves as an excellent proxy for individual migratory status and has been used as such or as a focal trait for the study of migration in monarchs in many studies [47, 59, 63, 66, 67, 69].

The variance in diapause induction in Queensland monarchs allows for a direct search for genetic associations with this trait and thus for an examination of the genetic underpinnings of the re-evolution of migration in Australian monarchs. Here, we used reduced representation sequencing to search for genes associated with migratory diapause in Queensland monarch butterflies. We found three strongly associated loci, one of which co-located with a spectrin beta chain protein *Karst*, which is involved in actin filament binding, is known to be expressed in silkworm ovaries, and is expressed during monarch diapause termination. This gene had not previously been identified as associated with migration in North American monarch butterflies, and we did not find that any previously identified migratory genes were associated with diapause in our study; however, using data we previously published from North American and Pacific monarchs we found evidence that the *Karst* SNPs associated with migration are present in North America.

## Methods

### Background

Although the ancestral range of monarch butterflies is believed to be in North and Central America, they have fairly recently expanded into South America and the Caribbean, and very recently to several locations throughout the Atlantic and Pacific [70–72]. In North America, the species is well known for its unique, multi-generational migratory life history wherein individuals dispersing northward in the spring do so over three to four generations and then return to their wintering grounds in a single step [73, 74].

In Southern Florida, the Caribbean, Central America, northern South America, and throughout most of their introduced range monarchs are non-migratory [75–77]. Residency in these populations appears to be a derived trait which has arisen multiple times after the species split from *Danaus erippus*, their closest extant relative [71]. The Pacific expansion of monarchs is relatively recent, with historical records indicating that they established first in Hawaii in the 1840s, likely as a result of individuals blown in during storm events that were able to survive on recently introduced milkweeds [55, 78]. Genetic evidence is consistent with an introduction to Hawaii during this time period [56]. As described above, they then spread across the Pacific and reached Australia in approximately 1871 [54] and were recorded migrating again in Australia sometime in the 1930s [57] (Fig. 1).

### Sample collection, incubation, and phenotyping

We collected 22 female monarch butterflies from Pinjarra Hills, Queensland, Australia (27°32′26.7″S, 152°54′22.7″E) between the 5th and 9th of July 2018. Individuals in all life stages were present at the field site during our sample collections, consistent with continuous, year-round breeding. We enclosed each of these 22 females individually on either *Asclepias curassavica* or *Gomphocarpus sp.* host plants found on-location and subsequently successfully collected eggs produced by 21 of them. While these eggs represent 21 total maternal families, each may consist of multiple groups of half-siblings since females may mate multiple times [79] and lay clutches with mixed parentage. We did not remove *Ophryocystis elektroscirrha* (a protozoan parasite) spores, which were uniformly present on our eggs (consistent with previous reports from this location [59]).

We then incubated all eggs using the same incubators and the “decreasing photoperiod” experimental scheme of Freedman et al. [59] in order to induce reproductive diapause and ensure that our data could be later pooled with that produced in the earlier study. Briefly, larvae were reared at a constant temperature of 28 °C under a photoperiod regime that declined from 14:10 L:D to 12:12 L:D over the course of 30 days (Δ4 mins/day). We released all male butterflies following emergence, since phenotyping diapause in male monarchs can be challenging due to the difficulty in extracting and weighing the ejaculatory duct, seminal vesicle, and accessory glands, which are likely the best indicator of male reproductive development [63]. This left us with a total of 164 females from 20 maternal families.

**Fig. 2:**
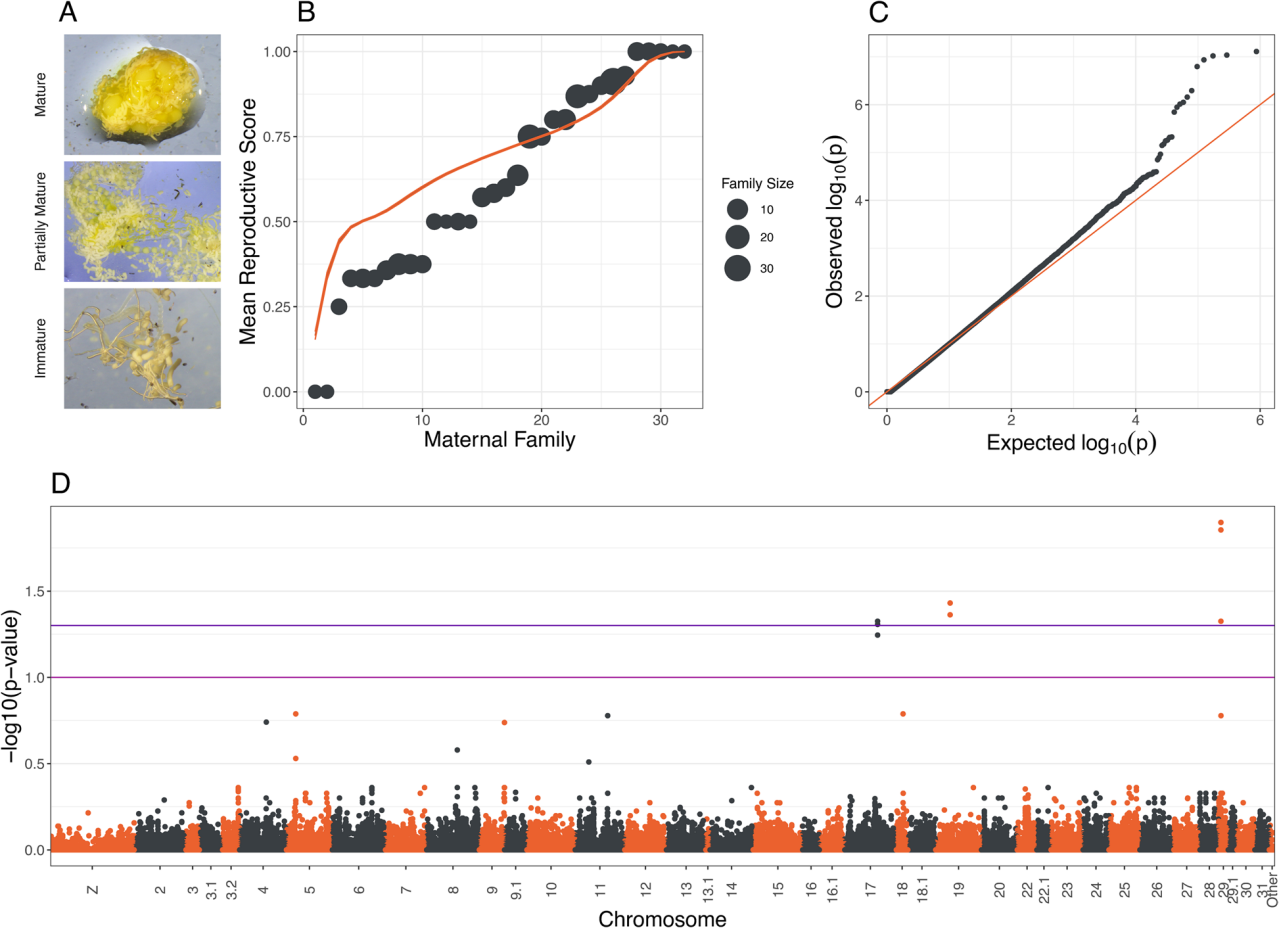
Genome-wide association results for diapause in Australian monarch butterflies. **A** Example egg development for the three stages used to phenotype adult females as reproductively mature, partially mature, and immature. **B** Average reproductive development scores for maternal families. Average scores were significantly more skewed than expected by chance (orange line). **C** Quantile–quantile plot for association results showing a few strong outliers but otherwise conformance to the expected distribution of *p*-values. **D** Manhattan plot showing the strength of association with reproductive status genome-wide. Values above the upper, blue line indicate significant associations after false discovery rate *p*-value adjustment

We assessed reproductive diapause by determining the degree of oocyte development in each of our adult female butterflies after 70 degree days of development, which is sufficient time for females to develop mature ovaries under normal summer conditions [80]. We observed that individuals fell into several defined bins: 97 out of 164 had fully developed, chorionated eggs (visible ridges along the exterior of the egg), while 67 females did not. Of these latter individuals, a few [11] had no yolk in their eggs whatsoever, but most had some degree of yolking. Since vitellogenesis generally only begins to a substantial degree after eclosion in monarchs [81], we classified individuals as either fully reproductively mature (with chorionated eggs), partially reproductively mature (with more than a tiny amount of yolking), or reproductively immature (with no or only very small amounts of yolking). Images of egg development in each individual are available online (see Data Availability, Fig. 2a). Wings and bodies from all individuals were preserved in dry coin envelopes or 95% ethanol, respectively. We note that while we refer to the postponement of reproductive development under otherwise suitable conditions as diapause, other authors refer to this process in monarchs as oligopause [82] or quiescence, since diapause implies an extended refractory period that we did not test for.

We supplemented these samples with preserved, dry butterfly wings from each of the “decreasing photoperiod” female individuals previously scored for reproductive development by Freedman et al. [59], constituting an additional 40 total female butterflies from 12 maternal families, which were reproductively scored by the authors as possessing chorionated, yolked, or unyolked eggs. Together, these two datasets contained 204 monarch butterflies from 32 maternal families (see Table 1).

**Table 1:** Counts of immature, partially mature, and mature female monarch butterflies reared and sequenced in each sampling year and the number of maternal families from which they came

Year	Immature	Partially Mature	Mature	Total Individuals	Maternal Families
2016	12	22	6	40	12
2018	11	56	97	164	20
Total	23	78	103	204	32

### Sequencing and genotyping

We removed and extracted DNA from a single leg from each of our samples collected in 2018 or from a wing base from each of the samples collected by Freedman et al. [59] using the magnetic bead protocol of Ali et al. [83]. We quantified the resulting DNA on a BioTek Instruments FLx800 Fluorescence Reader using Thermo Fisher Scientific Quant-iT PicoGreen dsDNA Reagent, then prepared Restriction Associated Digest (RAD) libraries using the Pst1 restriction enzyme according to Ali et al. [83]. We sequenced these 150 bp paired-end sequencing libraries using an Illumina Hi-Seq 4000.

We aligned the resulting raw sequence data to the “MEX_DaPlex” monarch butterfly genome assembly [84] using the mem algorithm of the Burrows-Wheeler Aligner [85]. We then filtered out PCR duplicates and improperly paired or poorly mapped reads using SAMtools [86]. For some downstream pedigree reconstruction, we then called genotypes using the ANGSD software [87] package with the following parameters: -doMajorMinor 1 (determine major and minor alleles using a genotype likelihood approach), -doMaf 2 (determine minor allele frequencies), -SNP_pval 1e-8 (keep only loci with a SNP *p*-value $\le 1\times {10}^{-8}$), -doGeno 4 (call genotypes), -doPost 2 (calculate genotype posterior probabilities using a uniform prior), -postCutoff 0.95 (keep only loci where the highest genotype posterior probability $\ge 0.95$), -minQ 20 (keep only loci with a sequencing quality $\ge 20$), -minMapQ 20 (keep only loci with a mapping quality $\ge 20$), -minInd 130 (keep only loci sequenced in at least 130 individuals), and -minMaf 0.05 (keep only loci with a minor allele frequency $\ge 0.05$).

### Statistical analysis

We first confirmed the parentage of our samples using the Colony2 program, assuming polygamous, random mating [88]. In order to determine the degree to which diapause status was biased between families, we fit a basic linear model in R version 4.2.2 [89] using phenotypic status as the response variable and maternal family as a fixed effect. Phenotypic status was coded as 0, 1, or 2 for reproductively immature, partially mature, or mature (as described above), respectively. We used an ANOVA test in R to determine if maternal ID significantly improved model fit. To determine the expected phenotypic distribution for each family sorted by mean reproductive rank (calculated from the individual 0, 1, 2 scores rescaled to 0, 0.5, and 1 for immature, partially mature, or mature, respectively such that a family with 100% reproductively mature individuals would have a score of 1) under a null distribution, we randomly permuted individuals between families 10,000 times and then ranked each family for reproductive score.

We conducted a Genome-Wide Association Study (GWAS) to determine if any loci were significantly associated with reproductive status. Since our data was generally of low coverage, we used a frequency test in ANGSD [87] based on genotype likelihoods rather than called genotypes and thus increased the effective number of loci we were able to analyze. We used the following parameters: -doMajorMinor 1, -SNP_pval 1e-12, -GL 1 (calculate genotype likelihoods using the SAMtools approach), -minQ 20, -minMapQ 20, -minInd 102 (50% of the individuals with called phenotypes), -minMaf 0.05, -doMaf 2 (assume a known minor allele), -doAsso 1 (do an association test using a frequency test), and -yquant, coding phenotypic status as above. Since our samples were composed of many groups of full and half-siblings and came from two genetically different years, we also used the -cov argument and supplied the first 20 principal components derived from PCAngsd [90], which we ran using the default parameters using genotype likelihoods derived from ANGSD using the same parameters plus -doGlf 2 to save genotype likelihoods. We calculated *p*-values for the resulting likelihood ratio test score and corrected those values using the false discovery rate approach of Benjamini and Hochberg [91] in R.

We constructed quantile–quantile and Manhattan plots of the resulting *p*-values using snpR [92] and identified candidate adaptive genes as those co-locating within 50 kb of any SNPs with a corrected $p\le 0.05$. Since the “MEX DaPlex” reference genome uses RefSeq gene IDs but most monarch studies use the monarch official gene set IDs, we used BLAST [93] to identify official gene set genes with protein sequences matching those of the RefSeq genes we identified via the built-in tool on MonarchBase [94].

To determine if diapause is part of a generalized migratory syndrome in Australia, we re-analyzed the connection between reproductive status and wing morphology in our 2016 samples. To determine if wing shape or size was correlated with reproductive status, we fit a pair of linear mixed effect models with either wing shape or size as response variables, the number of yolked oocytes as a fixed effect, and maternal family as a random effect using the R package nlme [95].

### Genotyping Karst in North American and other Pacific monarchs

To determine if the migration-associated variation we observed in the Australian monarchs was present in the North American and Pacific island populations from which the Australian population is derived, we used data which we previously published [56], which was produced using the same protocol and restriction enzyme as here. After downloading this data from the NCBI, we aligned and filtered it as described above. We then removed reads not located within 100 kb of *Karst* from both this dataset and sequencing data produced for this study using SAMtools [86] and called genotype likelihoods with ANGSD [87] for the region directly within *Karst* using the same options as above save for: -GL 2 (use the GATK method for calling likelihoods), -minInd 102 (keep only loci called in at least 102 individuals), and -doGlf 2 (produce a beagle formatted genotype likelihood file). We then imputed the *Karst* genotypes using beagle version 3.3.2 using the default options [96] in order to generate genotype calls. Given that poorly sequenced individuals and loci can bias downstream inference [97], we then removed calls for which the highest genotype imputation confidence was less than 95%, removed individuals and loci which were called in less than 60% of loci or individuals, respectively, and then identified and removed loci with minor alleles sequenced in less than five individuals with snpR [92]. We then calculated allele frequencies across all sampling locations for the six *Karst* loci our GWAS identified as associated with diapause in our Australian samples.

### Linear mixed modeling of diapause association with Karst

To determine the degree to which *Karst* explained variation in diapause status in the Australian monarchs, we constructed linear mixed models using the imputed genotypic data for the six diapause-associated *Karst* loci described above. Specifically, we used the lmerTest R package [98] to construct two linear mixed effect models with quantitative diapause status as the response variable and maternal and paternal IDs from Colony2 (as described above) as random effects. In one model, we also included additive genotypes for the six *Karst* loci as fixed effects. We then used a two-way ANOVA test in R compare the two models and determine if adding genotypic effects improved model fit.

## Results

### Phenotypic results and sequencing

Of the 204 total adult female monarch butterflies reared in both this study and by Freedman et al. [59], 103, 78, and 23 were classified as reproductively fully mature, partially mature, and immature, respectively. We obtained 396,512,006 total sequencing reads across all individuals, 99.4% of which mapped to the monarch reference genome and 60.7% of which were retained after filtering. From this, we called 179,735 and 437,259 SNP genotypes and likelihoods, respectively. Using the previously published North American and Pacific monarch samples [56], we called genotypes for a total of 685 imputed SNPs in *Karst* in 189 samples after filtering, of which 82 were from North America. 124 of the monarch samples collected for this study also passed filtering in the imputed *Karst* dataset.

### Genetic basis of diapause

**Fig. 3:**
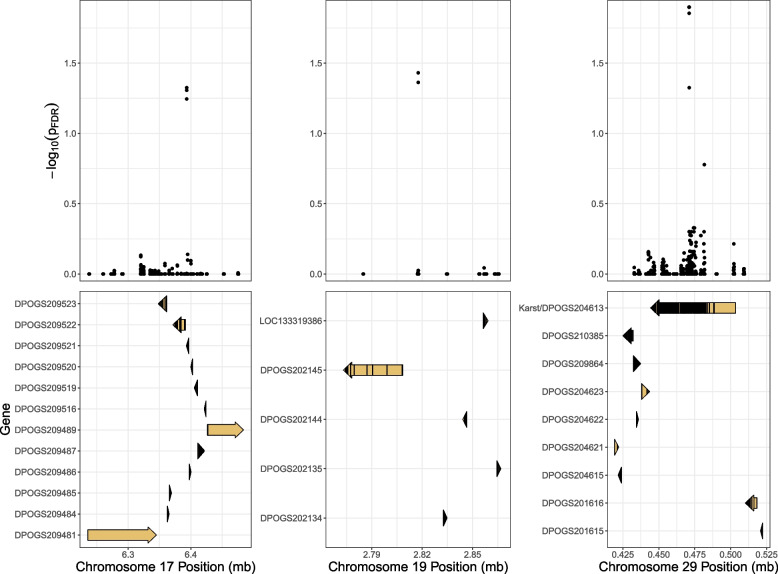
Distribution of genome-wide association test false-discovery rate adjusted *p*-values adjacent to each of the three outlier regions (top). Genes that are at least partially within 50kb of outlier SNPs are noted (bottom), with CDS regions highlighted in black. One gene (LOC133319386) had no match in the monarch official gene set, and so is instead listed with its RefSeq ID

Maternal family strongly improved model fit (ANOVA, $p<0.001$, see Fig. 2b). We identified three genomic regions which were significantly associated with reproductive development after false discovery rate correction, located on chromosomes 17, 19, and 29 (Fig. 2d). A total of 26 genes were within 50 kb of these regions (Table S1), one of which (LOC116776761/DPOGS204613/*Karst*) was located directly under the most significantly associated SNPs on chromosome 29 (minimum *p*-value = 0.013 (Fig. 3). No annotated genes were directly under the peaks of association on chromosomes 17 and 19 (Fig. 3). Imputed genotypes for the six significant *Karst* loci explained roughly 13.6% (marginal r^2^ = 0.135) of the variation in reproductive development after accounting for the effects of maternal and parental family. Given the tight physical linkage between these SNPs, all but one (SNP 1) were dropped from the model due to rank-deficiency. The relationship between diapause and additive genotype at that locus was highly significant (*p* < 0.0001), and the model with genotypic data was significantly preferred over the model without (two-way ANOVA, *p* < 0.0001).

We found that wing length was significantly negatively correlated with the number of yolked oocytes in Freedman et al.’s [58] earlier study ($p$ = 0.034) after accounting for maternal family. Wing shape was also negatively correlated with the number of yolked oocytes, but not significantly so after accounting for maternal family ($p$ = 0.26).

### Karst genotypes in North America and across the Pacific

**Fig. 4:**
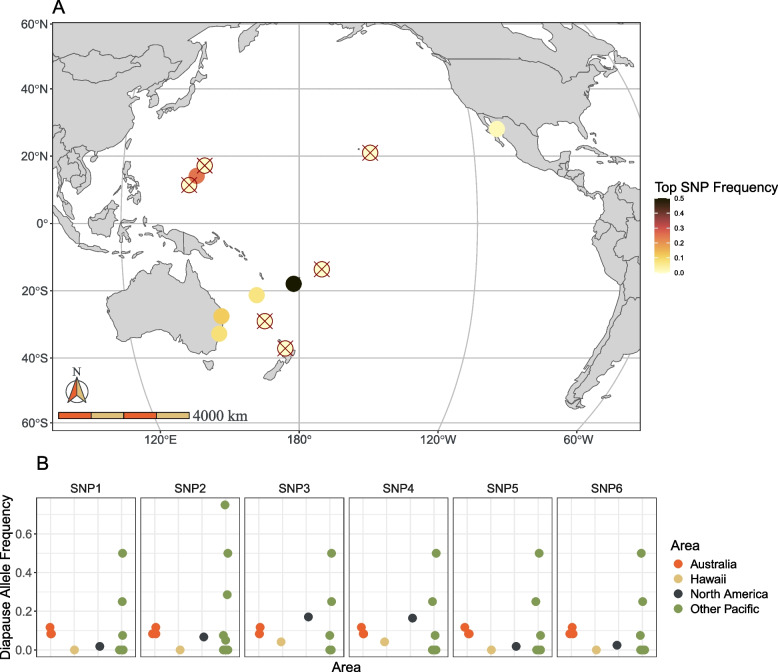
Distribution of diapause associated Karst alleles in North America and across the Pacific. **A** Frequencies for SNP 1, the most strongly associated with diapause in Australia, in North America, across the Pacific, and in Australia. Circles with a red “X” indicate locations with no detected diapause alleles. **B** Frequencies of diapause allele at all six of the significant Karst SNP loci in North America, Australia, Hawaii, and on other islands across the Pacific

The *Karst* SNPs associated with diapause in Australia were present in North America and across the Pacific at varying frequencies (Fig. 4). In general, the migration associated alleles varied strongly in frequency across the Pacific, were absent or very rare in Hawaii, and moderate in frequency in both Australia and in North America, with four out of the six more common in Australia and two more common in North America (Fig. 4b). The most strongly associated locus (SNP1) was more common in Australia than in North America, absent in Hawaii and several Pacific islands, and most abundant on Rota and Fiji (Fig. 1a).

## Discussion

### Evolution of diapause control in Australian monarchs

We found that a region on Chromosome 29 containing the gene DPOGS204613 (homologous to the gene *Karst* in *Drosophila* and hereafter referred to as such) was strongly associated with diapause initiation in monarch butterflies in Queensland, Australia (Fig. 3c), explaining roughly 14% of the variation in reproductive development in our study. According to the most recent annotation [84], *Karst* codes for a spectrin beta chain protein involved in actin filament binding. *Karst* has not been previously identified as associated with migratory life history variation in North American monarchs: specifically, *Karst* was not among the 536 genes located in regions of the genome found to be significantly associated with contrasts between migration and residency in North and South American, Pacific, and European monarch populations by Zhan et al. [71]. Nonetheless, we found that *Karst* alleles associated with diapause in Australia are present in North America at low frequencies (Fig. 4).

The involvement of *Karst* in migratory diapause initiation is perhaps not surprising. Juvenile hormone mediated actin contraction is known to be involved in oocyte formation [99], and mutations in *Karst* are known to cause serious issues during oogenesis in *Drosophila* [100]. The involvement of *Karst* in oocyte development is likely conserved in Lepidoptera: a predicted homolog of *Karst*, BGIBMGA012171, has been identified as an ovary-specific expressed protein in silkworms (*Bombyx mori*) [101]. More directly, while it does not overlap with any of genes identified as involved in photoperiodic responses by Iiams et al. [64], *Karst* has been observed to be differentially expressed during diapause termination in western North American monarchs [66]. Finally, *Karst* has been shown to be a key regulator of the *Hippo* pathway in *Drosophila*, in which RNAi knockouts of *Karst* have been shown to produce noticeable wing overgrowth [102]. Thus, variation at the *Karst* locus in monarchs could potentially contribute to differences in reproductive development as well as variation in wing size/elongation.

Given that we found that *Karst* alleles associated with diapause in Australia were present in North America, *Karst* migratory variance must have been either maintained as monarchs passed through repeated population bottlenecks during their Pacific expansion or secondarily re-introduced by immigrant individuals from North America (which would have likely been anthropogenic in origin). The latter seems unlikely given that (1) a secondary re-introduction of migratory monarch butterflies from North America would have resulted in the transfer of other migration associated and neutral genetic variation; (2) we did not find any of the previously known migratory associations in Australia; and (3) monarchs in Australia are generally very different from those in North America at neutral loci [56]. Regardless, it is surprising that *Karst* has not been previously identified to differ between migratory and non-migratory monarchs outside of Australia, suggesting that the mechanisms governing migration in monarchs worldwide are not yet fully understood.

In their 2014 study, Zhan et al. generally focused their discussion of monarch migratory genetics on Collagen IV $\alpha$−1, one of three genes they found which were strong outliers associated with migration. This gene is involved in muscle functioning in insects [103], and Zhan et al. hypothesized that divergence at this gene was driven by selection for increased muscle efficiency to facilitate long-distance migration. While they mentioned that the remaining significantly associated genes were enriched for the “morphogenesis, neurogenesis, and extracellular matrix/basement membrane” functional terms, they were not otherwise discussed (*Karst* does not have any of these functional terms). It is possible that *Karst* also contributes to diapause and migration in butterflies outside of Australia, but that the quantitative effect of other genes (a product of the effect sizes of the migratory alleles and their frequencies) dwarfs that of *Karst*, thus concealing the relative impact of the latter gene.

However, it does make some sense that variance in Collagen IV $\alpha$−1 would not be key to migratory life history variation in populations that are *newly* migratory. Variance in genes that code for traits such as muscle strength or endurance, such as Collagen IV $\alpha$−1, could make already migratory individuals more fit, but would not itself *cause* or *enable* migratory behavior. *Karst* and other genes that are associated with reproductive diapause may therefore be involved earlier in the evolution of migration, since individuals that do not delay their reproductive investment have much shorter lifespans and often cannot complete their full migration [60]. Genes that trigger migratory behavior or control orientation, navigation, and directed flight would also fall into this category to varying extents. That we found that *Karst* and not other genes were associated with diapause in Australia is therefore not surprising for a newly migratory population [104, 105].

Regardless, it is important to note that that *Karst* probably does not control diapause induction under declining daylength in Australian monarchs alone. We detected two other strong associations with diapause, but it is not clear to which genes these outliers correspond (Fig. 3a-b). There are a few potential reasons for this. First, it is possible that the causal variants are in transcription factor binding sites or other promoter regions which are not well annotated. Alternatively, the sequencing data in this study is relatively low in resolution, and we therefore may not have data for the actual causal variants with which our loci are in partial linkage disequilibrium. Higher resolution sequencing could again help clarify the causal genes for these regions. Additionally, while our study features many *individuals*, it features only 32 maternal families and thus far fewer independent samples. Our power is therefore limited, and it is entirely likely that we failed to detect many causal loci for diapause onset. Additional studies with larger sample sizes are therefore still needed to better understand the mechanisms underlying diapause induction in Australian monarch butterflies. Lastly, while the correlation between diapause and wing morphology which we observed does support the use of diapause as a proxy for the general migratory syndrome in Australian monarchs, future work examining correlations between *Karst* or other genes with different migratory traits, such as directed flight, could shed further light on the evolution of migration in Australian monarchs, as could sequencing and phenotyping monarchs from other migratory populations in Australia and New Zealand.

### Persistence of a migratory life history in Australian monarchs

Our work suggests that even after ceasing migration for hundreds of generations, monarchs may maintain genetic variation that underlies migratory plasticity, as they did in Australia despite the loss of allelic variance during multiple successive bottlenecks. This gives us some hope that the contemporary loss of migration that we have observed in many migratory species may be reversible over relatively short evolutionary timescales. At the very least, we can be assured that for monarchs in particular, migratory populations in Australia constitute a reservoir of migratory alleles that could potentially be tapped for North America if needed.

While monarchs may be able to quickly recover from the loss of migratory behavior, it is unlikely that this is the case for most other migratory species of conservation concern, particularly for vertebrates. Monarchs have had ample evolutionary opportunity to re-acquire migratory mutations and have been well equipped to maintain those they already held: monarchs have a generation time of approximately seven generations per year if continuously breeding [74], which means that monarchs in Australia went through roughly ~ 350 generations in the 50 or so years since they were first reported on the continent, and although they likely experienced a strong bottleneck initially, they were probably at a relatively large effective size for most of that time. Since the rate at which new mutations appear (and the rate at which standing variation is lost) in a population is proportional to both effective population size and generation time (i.e. “mutation-drift equilibrium") [106], monarchs have had a large opportunity space for the generation of new migratory alleles and the maintenance of old ones.

Additionally, the maintenance of ancestral North American migratory variance in Australian monarchs was probably only possible because selection was not actively acting against migration in the Pacific, where monarchs are not exposed to substantial seasonal changes in day length or average temperature and thus do not receive the primary cues thought to be associated with migration initiation. Environmentally triggered, phenotypically plastic migratory variation, therefore, is probably nearly neutral in the Pacific. In contrast, individually fixed migratory variance, such as wing morphology, was likely selected against across the Pacific, thus driving the observed, repeated decreases in wing size and length observed in newly non-migratory monarch populations [107]. Environmentally triggered migratory-associated genetic variation is therefore more likely to be maintained in non-migratory populations than that which underlies phenotypically fixed traits.

## Conclusions

This study suggests that reproductive diapause in Australian monarch butterflies is influenced by a novel genetic mechanism via the *Karst* gene. The previously identified variable migratory genomic regions were not associated with diapause in this population. Diapause associated variation at *Karst* is also present in North America and is therefore likely ancestral, maintained over successive bottlenecks during the species’ expansion across the Pacific. Our work is consistent with the hypothesis that genetic variance which enables migration, rather than that which bolsters the fitness of already migratory individuals, is more likely to be observed in recently evolved migratory populations.

## Supplementary Information

Supplementary Material 1.

## Data Availability

The raw sequencing data produced for this study are available from the NCBI under accession number PRJNA984345. The filtered genotypes and the scripts used to produce both them and this paper are available at https://github.com/hemstrow/aus_monarchs. The exact script used to produce the figures and tables for this paper from the genotypic data is available at https://github.com/hemstrow/aus_monarchs/blob/master/paper/Statistical_analysis.Rmd.
